# Beyond sequencing: re-visiting annotations for PJL as a test case

**DOI:** 10.1186/s13104-019-4508-5

**Published:** 2019-07-31

**Authors:** Waqasuddin Khan, Aisha Ghani, Muhammad Bilal Azmi, Safina Abdul Razzak

**Affiliations:** 1 Jamil-ur-Rahman Center for Genome Research, Dr. Panjwani Center for Molecular Medicine and Drug Research, International Center for Chemical and Biological Sciences, University of Karachi, Karachi, 75270 Pakistan; 2 Department of Biochemistry, Dow Medical College, Dow University of Health Sciences, Baba-E-Urdu Road, Karachi, 74200 Pakistan

**Keywords:** Variants, SNVs, InDels, PJL, Population-genetics

## Abstract

**Objectives:**

Current developments in sequencing techniques have enabled rapid and high-throughput generation of sequence data. However, there is a growing gap between the generation of raw sequencing data and the extraction of meaningful biological information. Variant annotation is a crucial step in the analysis of genome sequencing data. Incorrect or incomplete annotations can cause researchers to dilute interesting variants in a pool of false positives. We require consistent, accurate and reliable annotation of variants for making diagnostic and treatment decisions. Current annotation depends on the set of transcripts, and software used can be managed, with sufficient care, in the research context. Careful thought needs to be given to the choice of transcript sets and software packages for variant annotation in sequencing studies. In this project, the main objective is to analyze the genetic variants observed in Pakistani population data within the 1000 genomes project (1KGP).

**Results:**

We characterized only SNVs and InDels types of genetic variations, in total ~ 1.4 million variants. Besides this, we also annotated the genetic variants with multiple annotations tools, ANNOVAR and SnpEff and compared the differential results. Our population-specific catalogue will enhance future studies on the functional impact at protein level.

**Electronic supplementary material:**

The online version of this article (10.1186/s13104-019-4508-5) contains supplementary material, which is available to authorized users.

## Introduction

The phase3 of 1KGP [1] catalogues ~ 84.4 million genetic variations (frequencies of at least 1%) of 2504 individuals from 26 different world populations. However, many challenges still exist about the downstream analysis and interpretation of variants; most importantly researchers need to understand the functional consequences at the prime level of variant annotation. Pakistani sub-population (Punjabi in Lahore; PJL) data of South Asian (SAS) super-population represents genetic variation map of Pakistani origin. Careful variant annotation is a simple approach, usually seems to lead directly to the functional variants responsible for the differentiated phenotype or how it may affect the gene product if it occurs in the coding or non-sequence. In this study, efforts have been devoted to analyze the genetic variants of PJL sub-population as only two male Pakistani genomes are properly annotated [2, 3]. This will not only increase the information regarding variant annotations but also examine the choice of annotation protocol for further downstream analysis. We restricted our scope here to single nucleotide variants (SNVs) and short insertions and deletions (InDels). At the start of this study, emphasis has been put forward to count the genetic variants, supported by multivariate analysis to develop a model representing the divergence at chromosome level. For annotating and classification of genetic variants, we compared the annotation results of ANNOVAR [4] and SnpEff [5] while using the ENSEMBL transcript sets (ensGene database of ANNOVAR). Beyond issues specific to these particular transcript sets and software tools, we performed classical whole-genome annotation, although problems are yet to be solved. Due to the lack of skilled Bioinformaticians/next-generation sequencing (NGS) data analyst in Pakistan, very few to none publications are observed regarding the usage of PJL data. Keeping this in view, our laboratory has decided to further analyze the PJL data, and also to remind the research community about the forgotten PJL data of 6^th^ most populous country of the world. In future, the information generated by this work will be used to further explore the evolutionary history and genotype–phenotype variation relationship particular to this region of South Asia. Characterization of Pakistan-specific genetic variations could therefore significantly help in setting-up a reference/control panel that will contribute to the development of personalized medicine.

## Main text

### Methods

#### Retrieval of 1000 genomes project (1KGP) variant calls

Variant calls (final phase3 release) in the form of variant call format (*.vcf) files (version 4.2) were downloaded from the 1KGP website (ftp/mirror site: EBI FTP: ftp://ftp.1000genomes.ebi.ac.uk/vol1/ftp/; NCBI FTP: ftp://ftp-trace.ncbi.nih.gov/1000genomes/ftp/. Perl API scripts of VCFtools (v0.1.11) [6] were used to subset the vcf files. Population-specific vcf files (PJL sub-population vcf files) were generated by extracting 96 PJL samples (or individual) IDs considering only those sites that have alternative alleles in the PJL samples and skip any other sites that are all REF allele in PJL samples. BCFtools stats (version 1.1 + htslib-1.1, https://samtools.github.io/bcftools/bcftools.html) was used to count SNPs, InDels and ratio of Ts/Tv. SNPs densities were calculated in defined bins of 1 Mbs by SNPdensity output filtering statistics option of VCFtools.

#### Annotation of genomic variants

##### Selection of transcript set

ENSEMBL (version 83, December 2015) transcript set provides genome resources for chordate genomes with a particular focus on human genome data. ENSEMBL makes available substantial and diverse transcript information, including the Consensus Coding Sequence (CCDS) [7, 8], Human and Vertebrate Analysis and Annotation (HAVANA) (https://www.sanger.ac.uk/research/projects/vertebrategenome/havana/), Vertebrate Genome Annotation (VEGA) (Wilming, et al. [9]), ENCODE data [10] and the GENCODE gene and transcript sets [11]. 204,940 transcripts in ENSEMBL version 83 were used for annotations.

##### Variant annotations

Variant annotations were obtained using the software tool ANNOVAR (version 2015 Dec 14) and SnpEff (version 4.2 build 2015-12-15).

Annotations by ANNOVAR: ANNOVAR was used to functionally annotate genomic variants by two methods, (1) Gene-based annotation by ENSEMBL genes (ensGene) annotation database, and (2) Filter-based annotations, snp138, clinvar_20150629, cosmic68, cosmic70, 1000g2015aug_all annotation database were used. A broad interpretation of splicing regions was used for ANNOVAR annotations, so that all variants within six bases of an intron/exon boundary would fall into ANNOVAR’s splicing annotation category. ANNOVAR returns a single annotation for each variant. If there are several relevant transcripts for a particular variant, then ANNOVAR will return the annotation with the most severe consequence according to its rules of precedence.

Annotations by SnpEff: Variant annotations were also obtained using SnpEff based on GRCH37.75. As SnpEff returns all possible annotations for each variant (given the transcripts present at each variant’s location in the genome), we prioritized annotations by the consequence impact of the variant to make SnpEff annotation results directly comparable with those from ANNOVAR.

Statistical analysis and plotting were performed by different libraries loaded into R statistical package (version 3.2.1, https://www.r-project.org/).

### Results

Although, 1KGP data is available at http://www.internationalgenome.org/ but we compiled everything at one place related to PJL so that researchers and non-scientific community do not need to search from the scratch. PJL sub-population data has a total of 158 individuals but not all of them have the same kind of analysis. Individuals can be grouped on the basis of analysis and data collection, even some individuals are not sequenced at all (Additional file 1). Genetic variants of sequenced individuals are analyzed (number of SNVs and InDels, SNPdensitites, the frequency with which they occur, substitution types and along with their counts, and Ts/Tv ratio) selected on the basis of low-coverage WGS released in phase3 (Additional file 2: Figures S2–S6). The SNP counts of PJL sub-population are further compared with the 1KGP SNP counts (for this analysis, 1KGP have all SNP counts except PJL sub-population (Additional file 2: Table S1 and Figures S7–S9).

We commenced our investigation with the use of multiple annotation software in order to evaluate the influence of each algorithm on the resulting annotations. Here, we compared the variant annotation results of PJL sub-population as observed by ANNOVAR and SnpEff using the ENSEMBL transcript set (Additional file 2: Table S2). Primarily, we compared annotation terms categorized by both software. All exactly replicating categories are treated as individual affects, while particular categories in SnpEff are combined to compare against the broader ANNOVAR categories. We referred to an exact match when the annotations from two software are exactly equivalent. For example, both software annotate a variant as intronic or intergenic (Additional file 2: Table S3).

In total, 62,411 variants are annotated as exonic variants either by ANNOVAR or SnpEff (Additional file 2: Table S4). Of these, 23,678 (37.94%) variants are present in both tools. Interestingly, both annotation tools have good share of individual match rate (the number of annotated variants by either ANNOVAR, or SnpEff; could be said as private annotations), 61.5% for ANNOVAR and 98.64% for SnpEff. Intronic variants have the highest collective share of annotations (1,521,361) as identified by both tools. Almost all annotations found either in ANNOVAR or SnpEff have a higher concordance rate, 99.90% for ANNOVAR and 95.19% for SnpEff. Intergenic annotations also have the similar match rate, indicating the fact that both tools use similar approach to identify non-exonic variants. For splicing variants, 100% ANNOVAR match rate is observed for common variants; however, only 10.84% of those splice variants are annotated by SnpEff. Since SnpEff can predict much broad sequence ontology effects of splice variants, the greater number of splice variants provide more information of these locations. Likewise, upstream and downstream variants show an identical trend to splice variants with an overall exact match of 6% for both tools. Considering all annotation categories, ANNOVAR and SnpEff show a substantial amount of disagreement in annotating genetic variants, even when using the same transcripts. A comprehensive analysis of the data suggests that splicing, upstream, downstream, and non-coding exonic variants are present at a negligible concurrence. Further in-depth analysis will focus on the exonic versus intronic and intergenic variants, since these occupy the largest quantities of identified variants within the dataset. As we are not discouraging the use of either ANNOVAR or SnpEff, but the representation of annotated variants highlighted the emphasis of awareness of researchers that needs to meet while analyzing the annotated data. Our comparisons may highlight these discrepancies to some extent. The Sequence Ontology Project [12] helps us to minimize the effect of apparent differences of variant definitions (splice variants), eventually could improve the annotations for clinical usage. As per our experience, annotated variant with at least two tools should be associated with genes expression databases, such as GTEx [13] when considering functional assay validation on potential candidate/variants of interest. Variants with opposing, or missed annotations by one tool demands special handling [14].

## Limitations

Common population-specific genetic variants that are implicated in numerous diseases are non-randomly distributed throughout the genome, and make up the majority of varying nucleotides within human genomes. Pakistan, being the third world country, has no Genome-Wide Association Study (GWAS) ever launched that successfully pinpoint the association of genetic variants with disease or disease—related phenotypes. The individuals of PJL sub-population are mostly anonymous with no associated phenotype or medical data. However, this can help researchers to some extent to quickly cull out relevant genetic patterns and identify variants that lead to particular disorders. Even though, this classification will not inform us which variant is responsible for the increased risk of a disease, it will provide us the set of annotated suspects. As 1KGP PJL dataset has samples only from one geographical location of Pakistan (Punjab) that does not necessarily represent only one ethnicity (Punjabi), more or less these annotated genetic variants are not truly representatives of rich cultural diversity of Pakistan. Recently, some projects are started to coming out (https://www.nature.com/articles/nature22034) that will pave the pathway to enrich the Pakistani population data.

## Additional files

**Additional file 1.** Overview of the status of sequenced PJL samples, This file contains information regarding the sequencing, family data (trio statuses) and analysis of PJL samples.

**Additional file 2.** Analysis of PJL samples variants, This file includes the analytical description (with figures and tables) of PJL variants.

## Data Availability

The data described in this research note can be freely accessed as Additional files. The annotated VCF file is uploaded on figshare (10.6084/m9.figshare.6031916).

## Abbreviations

PJL: Punjabi in Lahore
SAS: South Asia
SNVs: single nucleotide variants
InDels: insertions and deletions
ANNOVAR: annotate variation
SnpEff: SNP effect
NGS: next-generation sequencing
GWAS: genome-wide association study
WGS: whole genome sequencing
WES: whole exome sequencing
1KGP: 1000 genomes project
