# Supplementary material for: Beyond sequencing: re-visiting annotations for PJL as a test case
Source: BMC Res Notes. 2019 Jul 31;12:467. doi: 10.1186/s13104-019-4508-5 (PMC6670121; doi:10.1186/s13104-019-4508-5)
Supplement: Supplementary file 2 — Additional file 2. Analysis of PJL samples variants, This file includes the analytical description (with figures and tables) of PJL variants. [file 13104_2019_4508_MOESM2_ESM.docx]

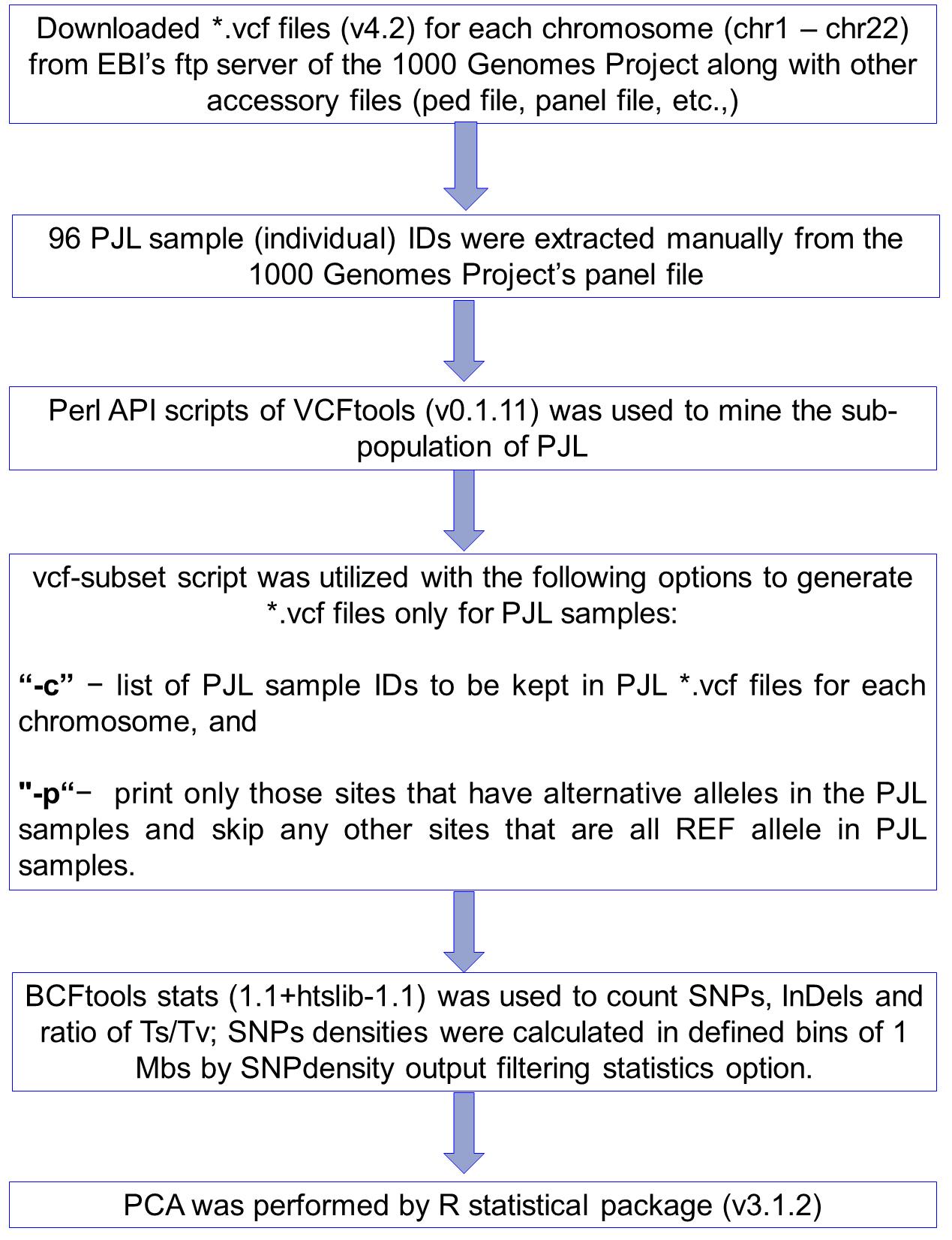


Annotations by ANNOVAR

Annotations by SnpEff

**Figure S1:** Workflow adapted performed during the project.


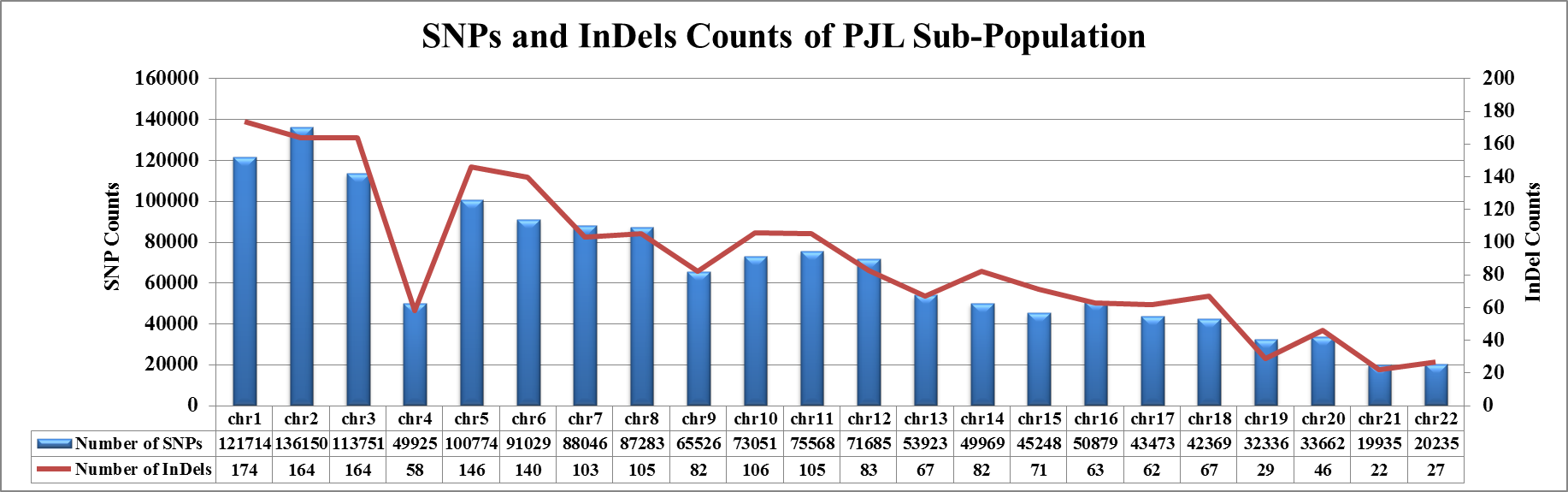


**Figure S2:** Genetic variants of PJL sub-population, categorized as counts of SNPs and InDels (see Materials and Methods section for full details, also applicable to Figures S3-S6).

The Figure S2 shows the sequential appearance of chromosomes with expected heterogenous distribution of variations which was further sub-categorized according to the number of SNPs and InDels counts. Genetic distribution of SNPs and InDels across the PJL sub-population genomes was varied in all the chromosomes. Among the 22 autosomal chromosomes, the minimum number of SNPs [19,935 (%1.36)], and InDels [22 (1.12%)] were detected in chr21, representing as the most conserved chromosome with respect to the PJL sub-population data. However, in case of maximum number of SNPs and InDels, chr2 [136,150 (9.28%)] and chr1 [174 (8.85%)] has the highest occurrence of SNPs and InDels, respectively.


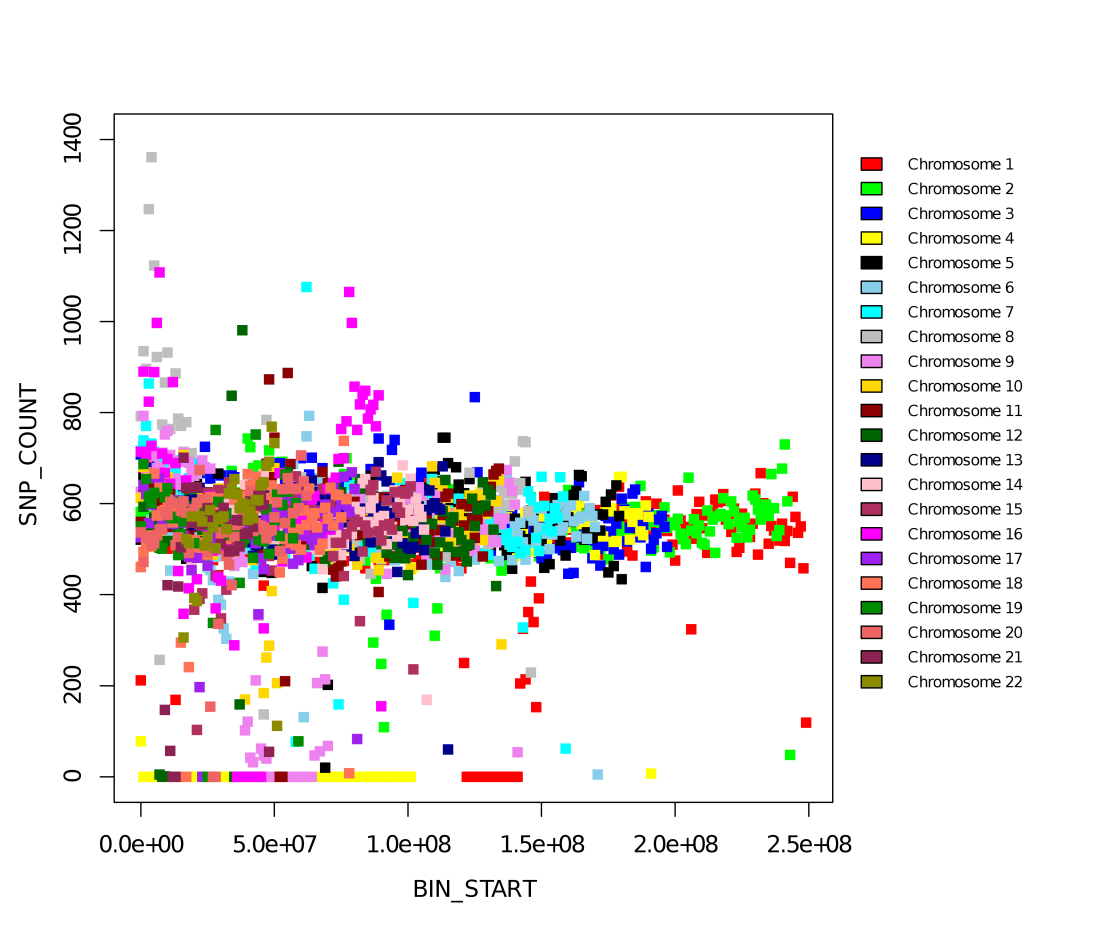


**Figure S3:**VCFtools’ perl script statistics module; SNPdensity was used to calculate the number and density of SNPs in bins of 1 Mbps in this case. There were some regions in chromosomes where no SNPs were observed.

Figure S3 shows an uneven distribution of SNPs and InDels across short arm to long arm of PJL sub-population was observed. SNPs were clustered with SNP density count range of approximately 20,000 – 40,000 SNPs (SNPs per Mbps). chr1 and chr2 has SNPs all over the chromosomes but chr8 has the highest number of SNP density (while chr4 has the least SNP density (see Table S1 below).


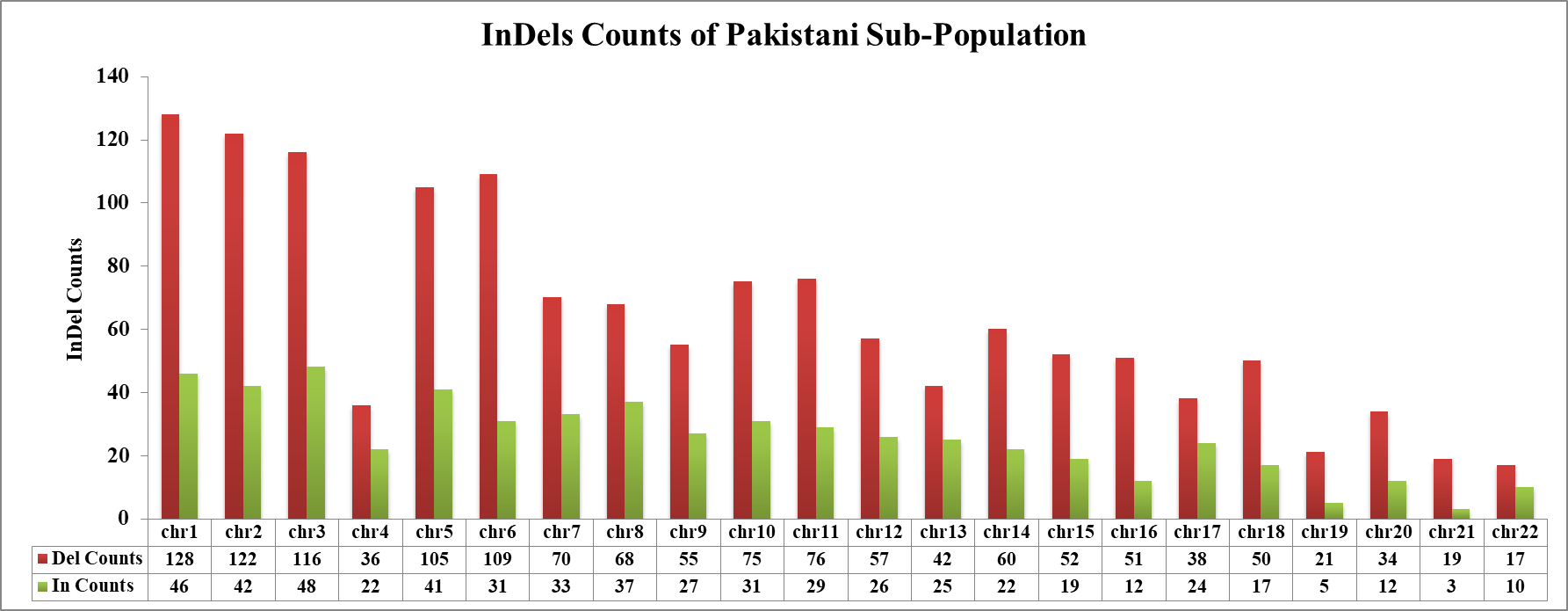


**Figure S4** InDels counts as observed in PJL sub-population.

Figure S4 shows the breakdown of insertions and deletions counts of PJL sub-population. chr21 has only three insertions (0.53%) while chr1 has the highest insertion counts [46 (8.19%)]. For deletions, chr22 [17 (1.21%)] and chr1 [128 (9.14%)] has the highest and lowest deletion occurrences.


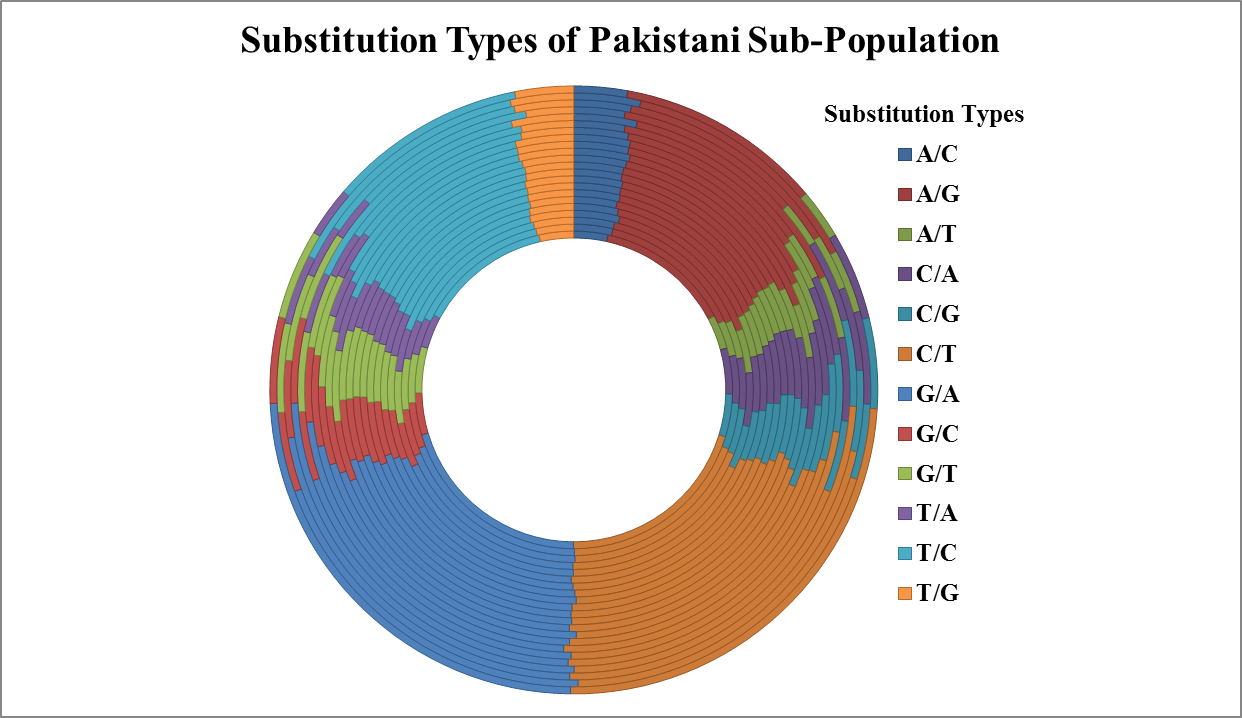


**Figure S5:**  Substitutions types as observed in PJL sub-population.

Figure S5 shows the regional substitution patterns in PJL genomes which was quite non-uniform. C/T substitution was occurring more frequently and shares the most part followed by G/A substitution.


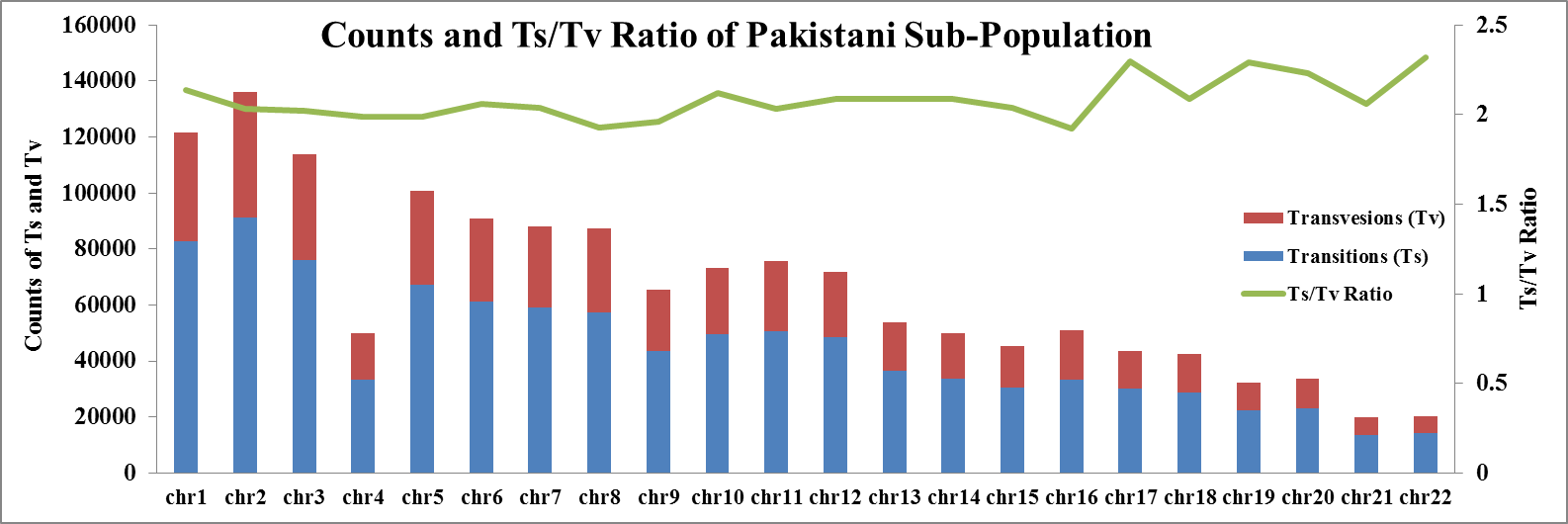


**Figure S6:** Counts and per chromosome Ts/Tv ratio as observed in PJL sub-population.

Figure S6 shows transitions (changes from A <-> G and C <-> T) were expected to occur twice as frequently as transversions (changes from A <-> C, A <-> T, G <-> C or G <-> T) across the entire genomes of PJL subpopulations. chr2 as the highest share of Ts with chr21 as the least with Ts. For highest Tv, chr2 has the higher ratio but for lowest Tv, chr21 has the least frequency of Tv. The Ts/Tv ratio remains constant till chr16 (averaging 2) but chr17-chr22 the ratio fluctutates to higher ratios. This represents that substitutions is located in the third base of a codon, that is, transversions which are much more likely to change the encoded amino acid. A transversion typically has a more marked effect as compared to transition because the third nucleotide codon position of the DNA, which to a large extent is accountable for the degeneracy of the code, is more tolerant of transition than a transversion: that is, a transition is more likely to encode for the same amino acid.

**(A)**


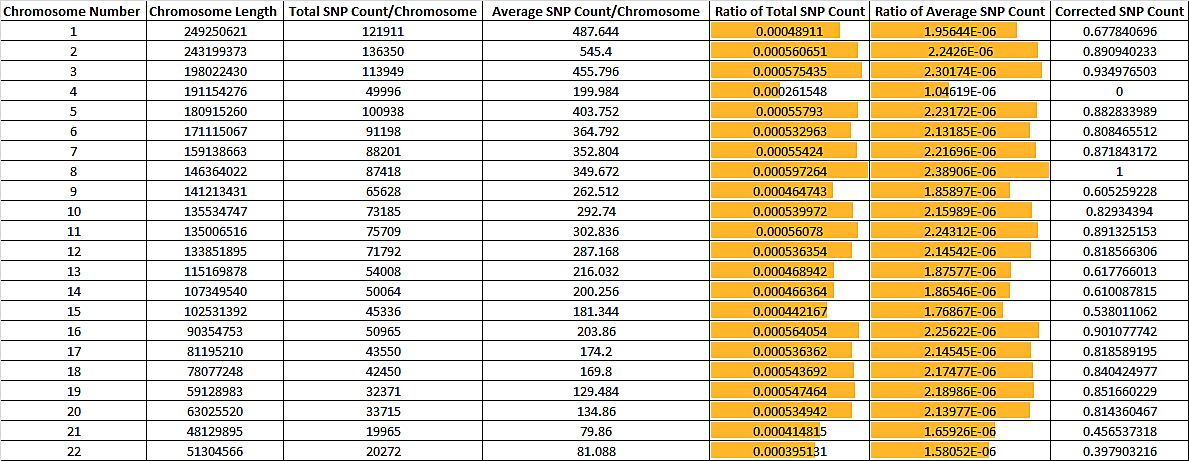


**(B)**


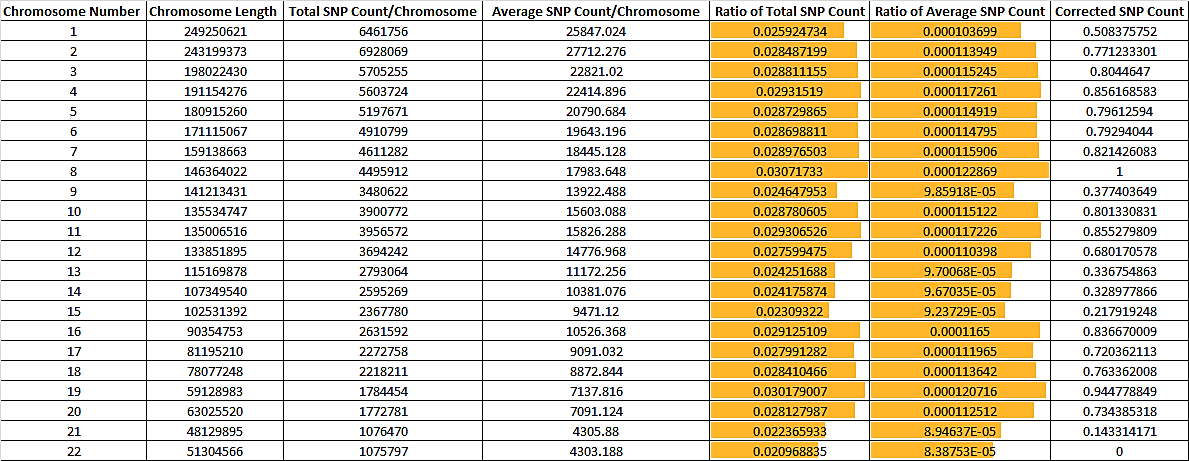


**Table S1:** Ratio calculated for each chromosome as per its length. The lower the ratio, the lower the variation observed for each chromosome (see Figure S7). For adjusting ratios on the scale of 0 - 1, corrected SNP counts were calculated by the following formula:

$$Corrected SNP Count= \frac{\left( Ratio of Total SNP Count- Minimum SNP Count \right)}{(Maximum SNP Count- Minimum SNP Count)}$$

The SNP counts of PJL sub-population were further compared with the 1KGP SNP counts (for this analysis, 1KGP have all SNP counts except PJL sub-population: 1KGP_-PJL_) (Table S1 and Figures S7-S9). Since the number of genetic variations are dependent on the length of chromosomes (the more the length of chromosomes, the greater number of variations are observed), this tends to generate a bias in terms of occurrence of variant counts. We corrected SNPs counts by using a normalization factor indicating chr4 as the least diverse chromosome in PJL sub-population (chr22 in case of 1KGP_-PJL_). This is in contrast to the 1KGP_-PJL_ corrected SNP counts where chr4 is ranked as second most variant rich chromosome (0.856) preceded by chr19 (0.944). The frequency of variations also remains the same for chr19 of PJL data with corrected SNP counts of 0.851. In case of highly variant chromosome, chr8 of both 1KGP_-PJL_ and PJL data has the corrected SNP count of 1.


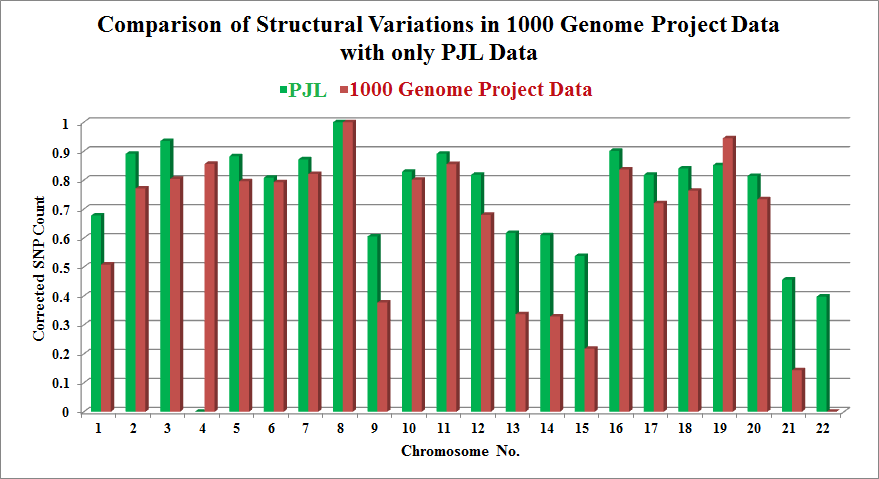


**Figure S7:** Corrected SNP counts were plotted with reference to the 1KGP data (see the description of Table S1).


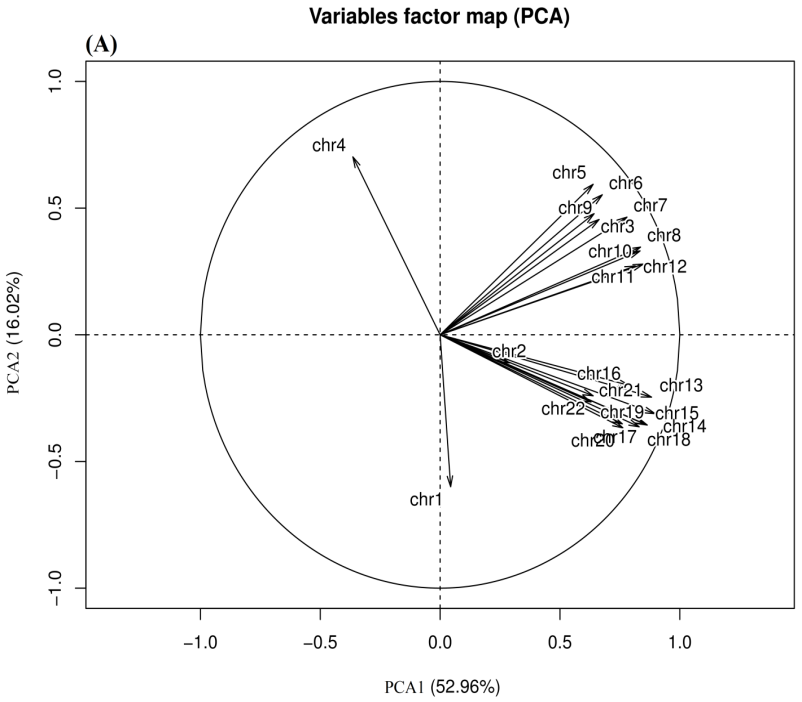

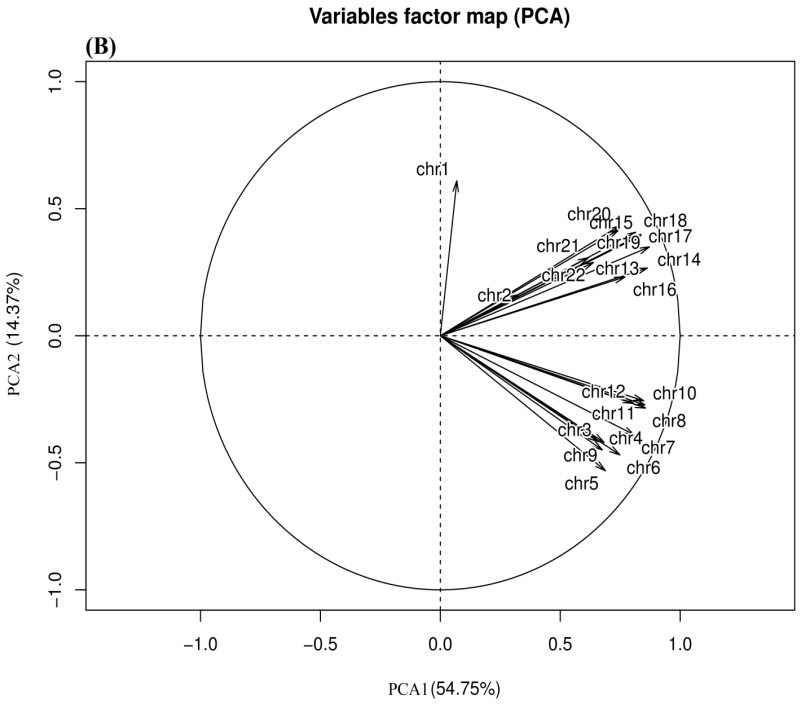


**Figure S8: Exploratory multivariate analysis of SNP densities by R package.** PCA of **(A)** PJL sub-population, and **(B)** 1000 Genomes Project. Both quantitative and qualitative variables, along with the inclusion of supplementary variables and observations were added to the analysis.


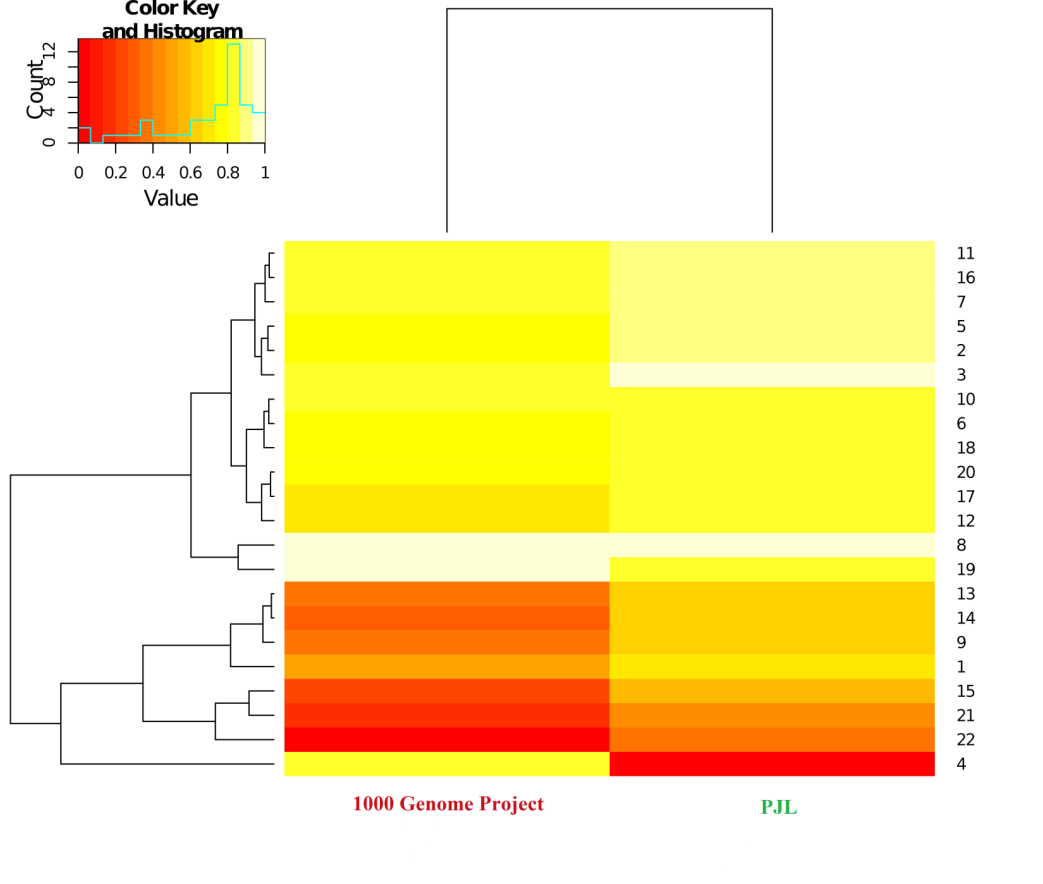


**Figure. S9:** Heat map of corrected SNP counts by R function. Heat map with colors were scaled according to the SNP densities (Transformed SNP densities: from orange to light yellow region; from low Z-scores to high Z-score values). Each column represents the chromosomes labelled on the vertical axis (right), and each row shows the SNP densities labelled on the horizontal axis (bottom) of the heat map. The dendrogram obtained with the hierarchical clustering analysis was displayed on the left. Clustering of chromosomes was achieved on the basis of SNP densities.

| **Annotations by ANNOVAR** | | | **Annotations by SnpEff** | | |
| --- | --- | --- | --- | --- | --- |
| **Annotation Type:** | **Count:** | **Percent:** | **Annotation Typ:e** | **Count:** | **Percent:** |
| Stop-gain | 378 | 0.03% | Stop-gain | 977 | 0.02% |
|  |  |  | Start Lost | 77 | 0.00% |
| Frameshift insertion | 1 | 0.00% | Frameshift Variant | 48 | 0.00% |
| Frameshift deletion | 11 | 0.00% |  |  |  |
| Stop-loss SNV | 31 | 0.00% | Stop-lost | 60 | 0.00% |
| Non-frameshift insertion | 1 | 0.00% | Inframe Insertion | 1 | 0% |
| Non-frameshift deletion | 4 | 0.00% | Inframe Deletion | 4 | 0% |
|  |  |  | Stop Retained Variant | 24 | 0% |
|  |  |  | Initiator Codon Variant | 11 | 0% |
| Nonsynonymous SNV | 15,003 | 1.02% | Missense Variant | 45,377 | 0.78% |
| Synonymous SNV | 7,975 | 0.54% | Synonymous Variant | 25,674 | 0.44% |
| Unknown | 219 | 0.01% |  |  |  |
| Exonic; Splicing | 27 | 0.00% | Exon Loss Variant | 371 | 0.01% |
| Intronic | 568,367 | 38.70% | Intron Variant | 338,0,901 | 57.72% |
| Downstream | 15,608 | 1.06% | Downstream Gene Variant | 534,032 | 9.12% |
| Intergenic | 633,053 | 43.10% | Intergenic Region | 651,141 | 11.12% |
|  |  |  | Intragenic Variant | 316 | 0.01% |
| ncRNA Exonic | 16,760 | 1.14% | Non Coding Exon Variant | 56,548 | 0.97% |
| ncRNA Exonic; Splicing | 12 | 0.00% | Non Coding Transcript Variant | 15 | 0% |
| ncRNA Intronic | 17,3941 | 11.84% |  |  |  |
| ncRNA Splicing | 93 | 0.01% |  |  |  |
| Splicing | 249 | 0.02% | Splice Acceptor Variant | 579 | 0.01% |
|  |  |  | Splice Donor Variant | 768 | 0.01% |
|  |  |  | Splice Region Variant | 11,045 | 0.19% |
| Upstream | 14,601 | 0.99% | Upstream Gene Variant | 516,959 | 8.83% |
| Upstream; Downstream | 1,029 | 0.07% |  |  |  |
| 3ˈ UTR | 16,365 | 1.11% | 3ˈ UTR Variant | 39,727 | 0.68% |
|  |  |  | 3ˈ UTR Truncation | 9 | 0% |
| 5ˈ UTR | 4873 | 0.33% | 5ˈ UTR Variant | 12,389 | 0.21% |
|  |  |  | 5ˈ UTR Premature Start Codon Gain Variant | 2,264 | 0.04% |
|  |  |  | 5ˈ UTR Truncation | 59 | 0.00% |
| 5ˈ UTR; 3ˈ UTR | 80 | 0.01% |  |  |  |
|  |  |  | TFBS Ablation | 125 | 0.00% |
|  |  |  | TF Binding Site Variant | 2,112 | 0.04% |
|  |  |  | Chromosome Number Variation | 1 | 0% |
|  |  |  | Disruptive Inframe Deletion | 6 | 0% |
|  |  |  | Protein Protein Contact | 2,159 | 0.04% |
|  |  |  | Sequence Feature | 573,655 | 9.79% |
|  |  |  | Transcript Ablation | 81 | 0.00% |
| **TOTAL** | 1,468,681 | | **TOTAL** | 5,857,515 | |

**Table S2:** Annotations of PJL genomic variants by ANNOVAR and SnpEff. Matched terms of both tools were categorized and colored.

| **Annotation Type:** | **Count of Common Annotations:** | **% of Common Annotations:** | **SnpEff:** | **SnpEff % Common with ANNOVAR:** | **% of total of SnpEff Annotations:** | **ANNOVAR:** | **ANNOVAR % Common with SnpEff:** | **% of total ANNOVAR Annotations:** |
| --- | --- | --- | --- | --- | --- | --- | --- | --- |
| Downstream Gene Variant | 15,594 | 1.07% | 227,076 | 6.87% | 11.51% | 15,608 | 99.91% | 1.05% |
| Frameshift Variant | 1 | 0.00% | 7 | 14.29% | 0.00% | 12 | 8.33% | 0.00% |
| Inframe Deletion | 0 | 0.00% | 3 | 0.00% | 0.00% | 4 | 0.00% | 0.00% |
| Inframe Insertion | 1 | 0.00% | 1 | 100.00% | 0.00% | 1 | 100.00% | 0.00% |
| Intergenic | 620,082 | 42.65% | 650,860 | 95.27% | 33.00% | 633,053 | 97.95% | 42.70% |
| Intron Variant | 741,553 | 51.00% | 779,053 | 95.19% | 39.50% | 742,308 | 99.90% | 50.07% |
| ncRNA Exon Variant | 16,759 | 1.15% | 39,737 | 42.17% | 2.01% | 16,772 | 99.92% | 1.13% |
| Nonsynonymous SNV | 14,967 | 1.03% | 15,207 | 98.42% | 0.77% | 15,003 | 99.76% | 1.01% |
| Splicing | 381 | 0.03% | 3,516 | 10.84% | 0.18% | 381 | 100.00% | 0.03% |
| Stop-gain | 376 | 0.03% | 384 | 97.92% | 0.02% | 378 | 99.47% | 0.03% |
| Stop-loss | 31 | 0.00% | 34 | 91.18% | 0.00% | 31 | 100.00% | 0.00% |
| Synonymous | 8,302 | 0.57% | 8,368 | 99.21% | 0.42% | 229,78 | 36.13% | 1.55% |
| Upstream Gene Variant | 14,589 | 1.00% | 219,119 | 6.66% | 11.11% | 14,601 | 99.92% | 0.98% |
| 3ˈ UTR | 16,417 | 1.13% | 22,124 | 74.20% | 1.12% | 16,445 | 99.83% | 1.11% |
| 5ˈ UTR | 4,952 | 0.34% | 6,676 | 74.18% | 0.34% | 4,953 | 99.98% | 0.33% |
| **Total** | **1,454,005** | 100% | **1,972,165** |  |  | **1,482,528** |  |  |

**Table S3:** Comparison of matched annotated terms of PJL genomic variants by ANNOVAR and SnpEff.

| **Type of Variants:** | **ANNOVAR + SnpEff** | **ANNOVAR** | **SnpEff** | **Exact Matches** | **Overall Exact Match Rate (%)** | **Grouped Matches** | **Overall Unnamed Match Rate (%)** | **ANNOVAR Match Rate (%)** | **SnpEff Match Rate (%)** | **Overall Match Rate (%)** |
| --- | --- | --- | --- | --- | --- | --- | --- | --- | --- | --- |
| Exonic | 62,411 | 38,407 | 24,004 | - | - | 23,678 | 37.94% | 61.65% | 98.64% | 52.12% |
| Intron Variant | 1,521,361 | 742,308 | 779,053 | 741,553 | 48.74% | - | - | 99.90% | 95.19% | 53.28% |
| Intergenic | 1,283,913 | 633,053 | 650,860 | 620,082 | 48.30% | - | - | 97.95% | 95.27% | 44.55% |
| Splicing | 3,897 | 381 | 3,516 | - | - | 381 | 9.78% | 100.00% | 10.84% | 0.84% |
| Upstream Gene Variant | 233,720 | 14,601 | 219,119 | 14,589 | 6.24% | - | - | 99.92% | 6.66% | 1.05% |
| Downstream Gene Variant | 242,684 | 15,608 | 227,076 | 15,594 | 6.43% | - | - | 99.91% | 6.87% | 1.12% |
| 3ˈ UTR | 38,569 | 16,445 | 22,124 | - | - | 16417 | 42.57% | 99.83% | 74.20% | 36.14% |
| 5ˈ UTR | 11,629 | 4,953 | 6,676 | - | - | 4952 | 42.58% | 99.98% | 74.18% | 10.90% |
| nc_exon_variant | 56,509 | 16,772 | 39,737 | 16,759 | 29.66% | - | - | 99.92% | 42.17% | 1.20% |
| **Total** | **3,398,184** | **1,465,756** | **1,932,428** | **1,391,818** | **40.96%** | 45428 | 1.34% | **94.96%** | **72.02%** |  |

**Table S4:** This table summarizes and compares the called annotations of ANNOVAR and SnpEff tools (while using the ENSEMBLE transcripts). The number of variants assigned to each sub-grouped category of annotation were listed when using either ANNOVAR or SnpEff [ANN+SnpEff], [ANNOVAR] alone, or [SnpEff] alone (see Table S2 and Table S3 for reference). The table also shows the quantity of variants that were [Exact Matches] such that both tools use the same annotation to categorize the variants and [Grouped Matches] such that multiple annotation types in SnpEff were combined to compare against the broader annotations of ANNOVAR. Columns E and G (column A is [Types of Variants]) display the overall count of match rate for the exact and grouped matches respectively, thus quantifying the number of overlapping annotations from the combined ANNOVAR or SnpEff variants (along with their percentages in columns F and H, respectively). Columns I & J provide the overall match rate in percentage for each of tools used in the analysis. Column K shows the overall match rate in percentage for each category.
